# Silent Worries: A Cross‐Sectional Study of School‐Going Adolescents' Unaddressed Health Concerns and Communication With Trusted Adults in Mongolia

**DOI:** 10.1002/hsr2.73025

**Published:** 2026-09-13

**Authors:** Ochirbat Batbold, Baasanjav Nachin, Tserendolgor Lkhagvasuren, Ser‐Od Erdenebileg, Christy Pu

**Affiliations:** ^1^ Medical School ACH Medical University Ulaanbaatar Mongolia; ^2^ Child and Adolescent Department National Mental Health Center Ulaanbaatar Mongolia; ^3^ Department of Research Mongolian Epidemiological Society Ulaanbaatar Mongolia; ^4^ Institute of Public Health National Yang Ming Chiao Tung University Taipei Taiwan

**Keywords:** adolescent health, health communication, help‐seeking behavior, mental health, undiagnosed disease

## Abstract

**Background and Aims:**

Adolescents may experience uncertainty about physical and mental health problems, and such uncertainty can negatively affect well‐being. In settings where access to appropriate services may be uneven, trusted adults can be an important source of support. This study investigated the prevalence of concerns regarding possible undiagnosed or untreated health conditions among adolescents in Mongolia and the likelihood that they would communicate these concerns to a trusted adult.

**Methods:**

A cross‐sectional study involving 4789 students from grades 7 to 12 in 13 schools across both urban and rural regions of Mongolia was conducted from June 1 to July 1, 2024. Data were collected through structured surveys administered during class time in a controlled environment. Adolescents reported their health status in relation to 12 mental and physical health conditions. Those who expressed concern regarding an undiagnosed or untreated condition were identified. The survey also assessed whether participants had discussed their concerns with a trusted adult. Logistic regression was used to analyze factors influencing help‐seeking behavior.

**Results:**

Perceived health concerns were most frequently reported for anxiety (12.11%) and depression (12.09%). Among physical health conditions, digestive disorders (11.23%) and heart disease (9.42%) were the most common perceived concerns. The likelihood of reporting communication with a trusted adult about any listed health concern varied across concern types but remained low overall.

**Conclusion:**

In Mongolia, adolescents often have significant concerns regarding undiagnosed or untreated health conditions. However, the likelihood that they will discuss these concerns with a trusted adult remains low. Addressing the barriers to communication between adolescents and adults is crucial for reducing the stress associated with health status uncertainty.

## 1 Introduction

Nearly 90% of adolescents worldwide live in low‐ and middle‐income countries with high fertility rates, making adolescent health a policy priority in these locations [1]. Adolescence is a critical period of physical, emotional, and psychological development, often accompanied by health uncertainties [2]. As adolescents undergo rapid physical and emotional changes, they may face unfamiliar health concerns, ranging from minor symptoms to serious worries [1].

Worrying about having an undiagnosed or untreated health condition can be a significant source of stress [3], which often stems from uncertainty regarding one's health status, leading to emotional discomfort and potential disruptions in daily life. Such concerns, even when not meeting the criteria for clinical health anxiety, can still negatively affect mental well‐being [4]. However, previous studies have not examined how often adolescents worry about having an undiagnosed health condition, whether they believe they have an untreated condition, or how frequently they discuss these concerns with a trusted adult.

Communication with trusted adults, such as parents or teachers, can play an important role in helping adolescents recognize health concerns and navigate available sources of support. However, in low‐ and middle‐income settings, this process may be less straightforward because adolescent services are often fragmented, poorly coordinated, and uneven in quality, and formal help‐seeking may be limited. In these contexts, trusted adults may function less as a direct link to the health‐care system and more as key sources of reassurance, guidance, and connection to locally available support [5, 6].

Bronfenbrenner's Ecological Systems Theory [7] can be used to clarify how trusted adults facilitate help‐seeking for physical and mental health concerns among adolescents. At the microsystem level, supportive relationships with adults create a safe environment in which adolescents feel comfortable discussing health concerns. At the mesosystem level, communication between these trusted adults (e.g., between parents and teachers) strengthens adolescent support, further encouraging help‐seeking. Although this behavior is also indirectly influenced by healthcare availability and cultural norms at the exosystem and macrosystem levels, immediate trust and guidance from familiar adults have the most direct role in shaping adolescent decisions to seek help.

Research suggests that connection with trusted adults is a key factor in promoting adolescent health and well‐being [8]. Existing studies on adolescent help‐seeking behavior have typically focused on mental health. A systematic review found that stigma and negative beliefs regarding mental health services and professionals were among the most prominent barriers to help‐seeking among adolescents [9]. However, in addition to mental health concerns, adolescents may experience chronic physical health problems, such as obesity [10, 11], diabetes [12, 13], cardiovascular diseases [14], hearing and vision problems [15, 16], or cancer [17]. Adolescents living with chronic conditions are at increased risk of experiencing mental health challenges, including anxiety and depression [17, 18, 19]. The relationship between physical and mental health deserves particular attention because untreated conditions can exacerbate symptoms, resulting in lower quality of life, difficulties in academic and social settings, and potential long‐term health complications [18]. Early intervention is crucial in breaking this cycle. Prompt management of the primary physical condition can prevent the onset of mental health concerns that may arise from physical ailments that remain untreated for a prolonged period.

The present study investigated how frequently adolescents communicate with trusted adults when experiencing either physical or mental health concerns. Mongolia provides a relevant setting for this question because it is a lower‐middle‐income country undergoing rapid urbanization while retaining strong family and community ties. In addition, access to adolescent‐responsive services may be uneven, particularly across rural and urban areas. These features make Mongolia a useful context for understanding how adolescents in lower‐resource settings perceive possible health problems and whether they turn to trusted adults for support.

We hypothesized that adolescents with different types of perceived physical or mental health concerns would differ in their likelihood of reporting communication with a trusted adult about any listed health concern, due to differences in perceived severity, uncertainty, and symptom recognition.

## 2 Methods

### 2.1 Survey Instrument

A Mongolian‐language questionnaire was designed and distributed. First, we reviewed the literature to identify common physical and mental health concerns experienced by adolescents. We also consulted specialists from the National Center for Mental Health, the only health care organization in Mongolia that focuses solely on mental health concerns. The head of the Youth Mental Health Department provided suggestions to refine the questionnaire and guided the selection of appropriate survey tools. Subsequently, six experts (two university professors, two school psychologists, and two classroom teachers) were invited to review the questionnaire and provide feedback to ensure construct validity. The preliminary questionnaire was administered to 10 students from grades 7 to 12, who also provided feedback. Adjustments were made on the basis of the feedback received and then the survey was finalized. The English version of the questionnaire is presented in Supplementary Material.

### 2.2 Participants

A cross‐sectional study involving 4,789 students from grades 7 to 12 across 13 schools in both urban and rural regions of Mongolia was conducted from June 1 to July 1, 2024. In Mongolia, compulsory education extends to 12 years, so the study population was drawn from the later stages of compulsory schooling. Specific cities/regions and participating schools were purposively selected to capture socioeconomic and geographic diversity across urban and rural Mongolia. Urban areas included Ulaanbaatar and Darkhan. Rural areas, namely, Zuunmod (Tuv Province), Mandalgovi (Dundgovi Province), and Choir (Govisumber Province), were chosen because the lifestyles of residents were more steeped in tradition and less affected by modernity (due in part to limited internet connectivity in these areas).

After the strata were determined, 2 to 3 schools from each stratum were randomly selected on the basis of population size of each province. Required data were collected from a list provided by the Mongolian Ministry of Education. Thus, a total of 13 schools were selected. The principal of each chosen school was informed about the study purpose, format, and content. All schools selected at this stage agreed to participate.

All students in grades 7 to 12 at each selected school were invited to participate in the study. Each student was given a written informed consent form for their primary guardian to sign and an additional form to sign themselves if they agreed to participate. Both forms explained the purpose and content of the study.

Students and their primary guardians provided consent for participation. A high participation rate of 96.5% was achieved, attributable to the support we received from the Mongolian Ministry of Education. Most students who did not participate in the study were absent from school when the survey was administered.

Data were collected through a structured process, in which all eligible students completed the online surveys during compulsory learning hours in computer classrooms. This approach ensured a controlled, distraction‐free environment and enabled students with limited internet and device access to participate. Teachers and members of our research team supervised the process to ensure that students completed the surveys independently; the average completion time was 15 to 20 min. All staff involved in this study were trained to maintain confidentiality and create a comfortable environment for participants. Students were informed that they could ask questions if they had any concerns. The students could see only their own computer screens. The survey was conducted anonymously, allowing the students to answer the questions freely.

### 2.3 Outcome Variables

Given that our goal was to examine whether adolescents who believed they were experiencing health concerns would speak to a trusted adult, the accuracy of self‐reported health conditions was not a focus of our study. Our survey included 12 health conditions known to be prevalent among adolescents: vision problems (“I feel I can't see clearly”), hearing problems (“I feel I can't hear properly”), digestive issues, heart conditions, respiratory problems, diabetes, asthma, cancer, musculoskeletal issues, autism, anxiety, and depression. Respondents were asked whether they had each condition and were required to select one of the following mutually exclusive responses: “No, I don't have this health problem,” “I don't know, but I don't care,” “I don't know, but I am worried,” “Yes, but I am not currently receiving treatment,” or “Yes, and I am currently receiving treatment.”

For each health condition, respondents who selected “I don't know, but I am worried” or “Yes, but I am not currently receiving treatment” were classified as adolescents with perceived health concerns. This was the primary group of interest in analyses of communication with a trusted adult. Respondents who selected “I don't know, but I don't care” were classified as not expressing concern and were grouped with the other non‐concern responses in the analysis. Our analyzes therefore focused on differences between adolescents who expressed concern about a possible health condition and those who did not.

Our survey measured help‐seeking behavior with the question “Do you ever talk to an adult (such as a teacher, parent, healthcare professional, or other adult you trust) when experiencing concerns about any of the above health conditions, such as to ask for advice or help?” Thus, this measure referred to communication with a trusted adult about any of the listed health concerns, rather than about a specific condition. The responses consisted of “Yes, always,” “Yes, sometimes,” and “No.”

### 2.4 Explanatory Variables

We included the following explanatory variables that may influence adolescent communication with a trusted adult when experiencing health concerns:

#### 2.4.1 Basic Demographic Characteristics

These included sex and age (level in school ranging from 7th to 12th grade).

#### 2.4.2 Socioeconomic Status

These were indicated by the father's education level (categorized as junior high or below, senior high, or college or above), mother's education level (junior high or below, senior high, or college or above), and the adolescent's self‐perceived household financial status (above average, average, or below average), which was used as a subjective indicator of household economic position rather than an objective measure of household income or assets.

#### 2.4.3 Other Caregiver Characteristics

These were whether the primary caregiver was the child's parent or parents (yes/no) and whether parents were married (categorized as married, divorced, or other).

#### 2.4.4 Other Factors

These included area of residence (categorized as rural or urban), self‐perceived academic ranking (categorized as top 10%, 11%–30%, 31%–50%, or below 50%), current smoking status (yes/no), recreational alcohol consumption within the past year (yes/no, excluded cooking purposes), and self‐rated general health status (categorized as excellent, very good, good, fair, or poor).

### 2.5 Statistical Analysis

Descriptive statistics were used to summarize participant characteristics and response frequencies. Differences in participant characteristics between rural and urban areas were assessed using chi‐square tests for categorical variables and Student's t test for continuous variables. For the question on adolescent communication with a trusted adult regarding health concerns, the responses “Yes, always” and “Yes, sometimes” were combined. For each of the 12 health conditions, we created a binary indicator for perceived health concern (1 = “I don't know, but I am worried” or “Yes, but I am not currently receiving treatment”; 0 = all other responses). Logistic regression models were then used to examine associations between explanatory variables and communication with a trusted adult. Odds ratios (ORs), 95% confidence intervals (CIs), and predicted probabilities were calculated from the regression models. All tests were two‐sided, and a *p* value < 0.05 was considered statistically significant. All analyses were performed using Stata 18 [20].

## 3 Results

Table 1 presents the participants' demographic characteristics. A total of 4789 adolescents were included in our analysis. Of them, 57.4% were girls. The mean age of study cohort was 15.02 ± 1.72 years. No significant differences were observed in sex or age between adolescents residing in rural areas and those residing in urban areas. A higher proportion of parents from urban areas had a college degree or above compared with those from rural areas. However, urban and rural areas of residence did not significantly differ in terms of perceived household financial status. The self‐reported alcohol consumption rate among adolescents was significantly higher in urban areas than in rural areas.

**Table 1:** Demographic characteristics of adolescent participants from five Mongolian provinces (*N* = 4789).

	Total		Rural		Urban		*p* value^1^
	*N*	%	*n*	%	*n*	%	
	4789		2657		2132		
Sex						
Male	2040	42.60	1146	43.13	894	41.93	0.404
Female	2749	57.40	1511	56.87	1238	58.07	
Age (mean, SD)^2^	15.02	1.72	15.02	1.66	15.01	1.79	0.871
Primary guardian							
Parent	4079	85.17	2181	82.09	1898	89.02	< 0.001
Grandparent(s)/other relative(s)/other	710	14.83	476	17.91	234	10.98	
Parent marital status							
Married	4119	86.01	2274	85.59	1845	86.54	0.345
Divorced/other	670	13.99	383	14.41	287	13.46	
Father's education level							
Junior high school or below	851	17.77	583	21.94	268	12.57	< 0.001
Senior high school	2161	45.12	1209	45.5	952	44.65	
College or above	1777	37.11	865	32.56	912	42.78	
Mother's education level							
Junior high school or below	411	8.58	274	10.31	137	6.43	< 0.001
Senior high school	1861	38.86	1087	40.91	774	36.3	
College or above	2517	52.56	1296	48.78	1221	57.27	
Perceived financial status							
Below average	254	5.3	144	5.42	110	5.16	0.693
Average	2062	43.06	1130	42.53	932	43.71	
Above average	2473	51.64	1383	52.05	1090	51.13	
Perceived academic performance							
Top 10%	1061	22.15	645	24.28	416	19.51	< 0.001
Top 11%–30%	1122	23.43	627	23.6	495	23.22	
31%–50%	1339	27.96	733	27.59	606	28.42	
Below 50%	1267	26.46	652	24.54	615	28.85	
Smoking status							
Yes	316	6.6	166	6.25	150	7.04	0.275
No	4473	93.40	2491	93.75	1982	92.96	
Alcohol consumption							
Yes	327	6.83	154	5.80	173	8.11	0.002
No	4462	93.17	2503	94.20	1959	91.89	

^1^ Statistical analyses: the *t* test was performed for continuous variables, whereas the chi‐square test was performed for categorical variables.

^2^ SD, standard deviation.

**Figure 1:**
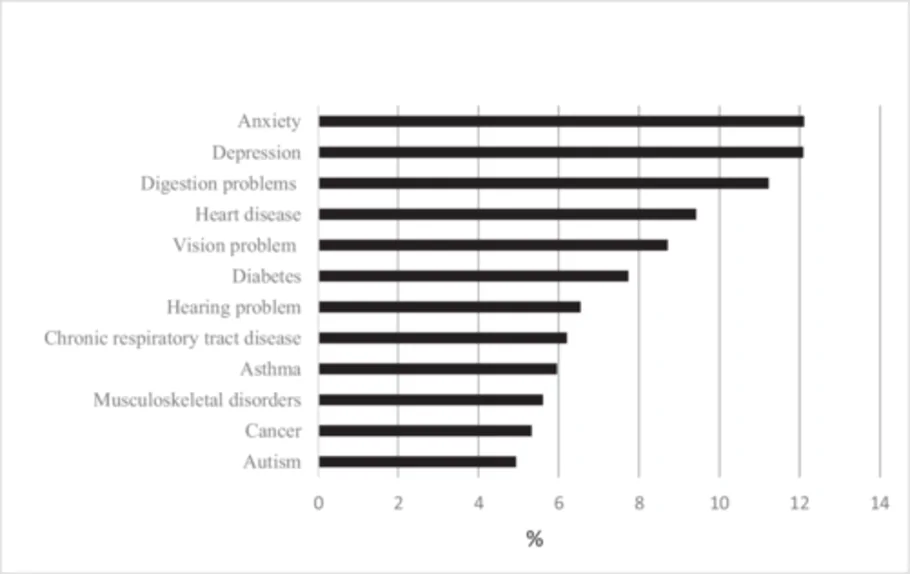
Percentage of adolescents who responded “I don't know, but I am worried” to health condition questions. Figure legend: Percentages are based on the entire sample (*n* = 4789) and indicate the proportion of adolescents who selected the response option “I don't know, but I am worried” for each health condition.

Figure 1 illustrates the percentage of adolescents who responded to health condition questions with “I don't know, but I am worried,” among which anxiety (12.11%) and depression (12.09%) were the two conditions most frequently reported. Among physical health conditions, respondents worried most about digestive disorders (11.23%) and heart disease (9.42%). Additionally, 5.32% of respondents were concerned that they may have cancer. Unlike the other health conditions listed, autism is typically diagnosed at a young age (at an average age of 5 years) [21]. However, approximately 5% of respondents were worried they may have undiagnosed autism.

**Figure 2:**
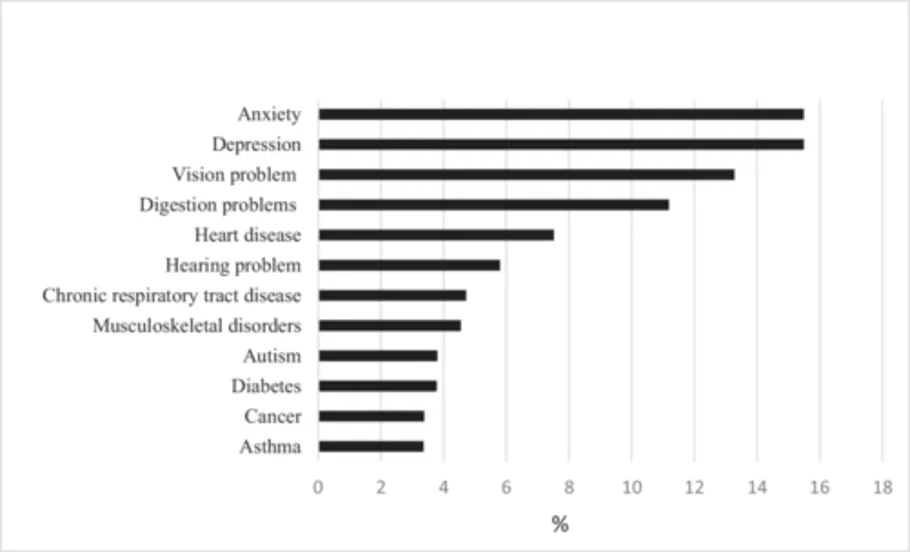
Percentage of adolescents who responded “Yes, but I am not currently receiving treatment” to health condition questions. Figure legend: Percentages are based on the entire sample (*n* = 4789) and indicate the proportion of adolescents who selected the response option “Yes, but I am not currently receiving treatment” for each health condition.

Figure 2 shows the percentage of adolescents who responded to health condition questions with “Yes, but I am not currently receiving treatment.” Approximately 15.5% reported untreated depression, and a similar percentage reported untreated anxiety; these were the highest percentages among all health conditions listed. The next most frequently reported conditions were untreated vision problems (13.3%), digestive disorders (11.2%), and heart disease (7.52%). Additionally, 3% of respondents self‐reported untreated cancer. Supporting Information Table S1 shows the combined prevalence of adolescents with perceived health concerns across the 12 health conditions.

Table 2 provides estimated ORs for explanatory variables that may have influenced the decision to communicate with trusted adults regarding health conditions. Adolescent boys had a significantly lower likelihood of having spoken with a trusted adult compared with adolescent girls (OR: 0.668; 95% confidence interval [CI]: 0.570–0.784). Compared with participants in grade 7, those in grades 8, 10, and 11 were more likely to have spoken with a trusted adult. Adolescents residing in urban areas were less likely to have spoken with a trusted adult than were those residing in rural areas (OR: 0.803; 95% CI: 0.690–0.933). Current smoking status exerted no significant effects on the likelihood of communication, whereas recreational alcohol consumption was associated with an increased likelihood of communication (OR: 1.896; 95% CI: 1.437–2.502).

**Table 2:** Factors influencing the communication between adolescents and trusted adults regarding health concerns (*N* = 4789).

	Bivariate				Multivariate			*p* value
	Odds ratio	95% confidence interval		*p* value	Odds ratio	95% confidence interval		
Sex (reference = female)								
Male	0.550	0.476	0.635	< 0.001	0.668	0.570	0.784	< 0.001
Grade (reference = grade 7)								
Grade 8	1.643	1.284	2.103	< 0.001	1.529	1.178	1.983	0.001
Grade 9	1.305	1.031	1.651	0.027	1.262	0.985	1.616	0.066
Grade 10	1.878	1.479	2.384	< 0.001	1.459	1.130	1.884	0.004
Grade 11	1.610	1.276	2.031	< 0.001	1.354	1.056	1.736	0.017
Grade 12	1.441	1.105	1.880	0.007	0.988	0.736	1.326	0.936
Guardian (reference = parents were not primary guardians)								
Parents	0.945	0.782	1.141	0.557	1.052	0.854	1.296	0.636
Father's education level (reference = junior high school or below)								
Senior high school	0.857	0.709	1.035	0.109	0.905	0.726	1.127	0.372
College or above	1.001	0.826	1.212	0.995	1.067	0.839	1.356	0.598
Mother's education level (reference = junior high school or below)								
Senior high school	1.030	0.796	1.333	0.822	1.030	0.765	1.388	0.845
College or above	1.049	0.816	1.349	0.710	1.044	0.770	1.415	0.783
Self‐reported financial status (reference = below average)								
Average	0.834	0.616	1.130	0.242	1.008	0.723	1.406	0.962
Above average	0.910	0.674	1.228	0.538	1.099	0.787	1.533	0.580
Parental marital status (reference = divorced or other)								
Married	1.135	0.938	1.374	0.194	0.956	0.774	1.179	0.671
Area (reference = rural)								
Urban	0.805	0.702	0.924	0.002	0.803	0.690	0.933	0.004
Self‐rated health status (reference = excellent)								
Very good	1.255	1.015	1.551	0.036	1.069	0.855	1.335	0.559
Good	1.479	1.200	1.824	< 0.001	1.068	0.851	1.340	0.570
Fair	2.055	1.636	2.580	< 0.001	1.257	0.975	1.620	0.078
Poor	2.223	1.377	3.588	0.001	1.014	0.587	1.751	0.960
Current smoking status (reference = no)								
Yes	1.451	1.127	1.868	0.004	1.312	0.978	1.760	0.070
Recreational alcohol consumption (reference = no)								
Yes	2.211	1.748	2.797	< 0.001	1.896	1.437	2.502	< 0.001
Self‐reported academic performance (reference = top 10%)								
Top 11%–30%	0.947	0.771	1.162	0.601	0.870	0.698	1.083	0.213
31%–50%	1.055	0.869	1.281	0.586	0.974	0.791	1.199	0.805
Below 50%	1.164	0.959	1.413	0.125	1.024	0.830	1.264	0.826
Self‐reported health concerns (reference = no)								
Asthma	1.544	1.246	1.915	< 0.001	0.857	0.603	1.218	0.390
Chronic respiratory diseases	2.005	1.650	2.435	< 0.001	2.141	1.558	2.944	< 0.001
Musculoskeletal disorders	1.639	1.335	2.013	< 0.001	0.929	0.671	1.286	0.656
Heart diseases	2.478	2.107	2.915	< 0.001	1.641	1.308	2.059	< 0.001
Diabetes	1.508	1.238	1.837	< 0.001	0.708	0.522	0.959	0.026
Cancer	1.328	1.059	1.667	0.014	0.573	0.377	0.873	0.010
Digestive disorders	2.747	2.367	3.188	< 0.001	1.667	1.365	2.037	< 0.001
Autism	1.154	0.914	1.456	0.229	0.326	0.211	0.505	< 0.001
Depression	2.634	2.284	3.038	< 0.001	1.435	1.137	1.812	0.002
Anxiety	2.495	2.163	2.877	< 0.001	1.121	0.886	1.417	0.342
Hearing problems	1.764	1.462	2.128	< 0.001	1.087	0.826	1.431	0.550
Vision problems	2.975	2.562	3.454	< 0.001	2.259	1.876	2.720	< 0.001

**Figure 3:**
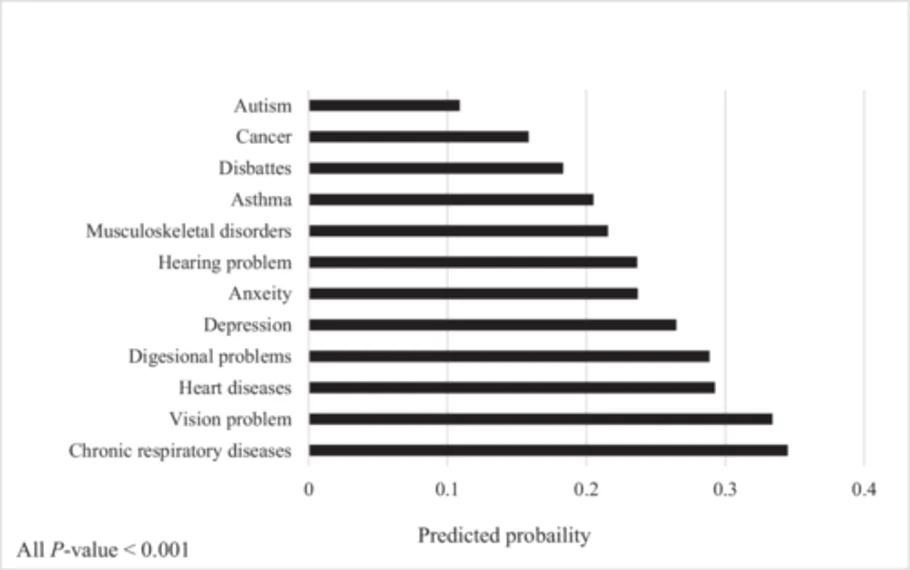
Communication probability by health condition. Figure legend: Predicted probabilities were estimated from the logistic regression model. For each of the 12 health conditions, adolescents were classified as having a perceived health concern if they selected either “I don't know, but I am worried” or “Yes, but I am not currently receiving treatment.” The outcome was whether the adolescent reported having talked to a trusted adult about any of the listed health concerns.

In Figure 3, we estimated the probability of reporting communication with a trusted adult among adolescents with perceived concern for each health condition, based on the logistic regression shown in Table 2. Because the help‐seeking outcome referred to talking to a trusted adult about any of the listed health concerns, these probabilities should not be interpreted as condition‐specific disclosure. Rather, they indicate the likelihood of general communication with a trusted adult among adolescents who expressed concern about each condition.

## 4 Discussion

Our study investigated the prevalence of concerns regarding undiagnosed or untreated health conditions among adolescents and the likelihood that they would communicate with a trusted adult as a result. We hypothesized that this likelihood would vary depending on the type of health condition. Our key findings are as follows: (1) A high proportion of adolescents reported being worried about having undiagnosed or untreated anxiety and depression compared with other conditions, and the frequency of self‐reporting differed considerably across conditions; (2) The likelihood of reporting communication with a trusted adult differed across groups defined by perceived concern for different health conditions. However, because the help‐seeking measure referred to talking to a trusted adult about any of the listed health concerns rather than about a specific condition, these findings should not be interpreted as indicating whether adolescents disclosed that particular condition; and (3) The overall likelihood of an adolescent discussing health concerns with a trusted adult was low.

We found that potential undiagnosed or untreated anxiety and depression were the most common health concerns among adolescents. Although their symptoms may not be severe enough to meet the criteria for a clinical diagnosis, such concerns can still indicate underlying emotional difficulties that could benefit from attention or intervention. Our findings reinforce the relevance of Bronfenbrenner's ecological systems theory in explaining adolescents' help‐seeking behaviors. The low likelihood of help‐seeking for even serious conditions may reflect microsystem limitations, such as weak or underutilized support from parents or teachers, and points to broader mesosystem or macrosystem factors, such as stigma or cultural attitudes that discourage open dialog. These findings highlight the need to strengthen immediate support systems and optimize the wider social environment surrounding adolescents.

Early recognition of health concerns is crucial for addressing mood problems before they escalate, improving mental well‐being, and preventing the development of more serious conditions. For adolescents, communication with a trusted adult at this stage fosters early intervention. Depression and anxiety can lack outward physical symptoms, and the need for treatment may thus go unrecognized or unexpressed [22]. Furthermore, stigma surrounding mental health may deter adolescents from seeking help, despite high levels of concern regarding mental health conditions [23]. Early recognition and appropriate interventions are crucial for addressing these concerns and improving treatment rates. Overall treatment rates for mental health conditions in children and adolescents, particularly for depression and anxiety, remain low [22], which is consistent with our findings.

It is also important to note that whether disclosure leads to timely intervention in Mongolia may depend on the availability, accessibility, and acceptability of adolescent‐responsive services. Although primary health care in Mongolia is theoretically free, its utilization varies between rural and urban areas and across population groups [24].

Although previous studies have indicated that children and adolescents are reluctant to seek support for emotional difficulties from trusted adults [9, 25], we found that adolescents are also reluctant to disclose physical health concerns, which has several plausible explanations. Adolescents may hesitate to discuss health concerns with trusted adults due to fear of overreaction, worrying that adults might take drastic actions or advocate for unwanted medical interventions, or that they may distrust the medical provider [26]. At the other extreme, adolescents may be afraid that adults will not take their concerns seriously [9, 27, 28]. Additionally, they may experience self‐doubt regarding whether their symptoms are serious enough to discuss [29]. The desire for independence can also cause adolescents to feel that handling health matters on their own is part of growing up and demonstrating maturity [30].

Although studies have suggested that depression, anxiety, and other mental health concerns are underreported due to stigma or symptom misrecognition [9, 25], these conditions were the most frequently reported health concerns in our sample. This finding may seem counterintuitive but is attributable to the structure of our survey. We did not directly ask adolescents to confirm or deny having a mental health concern in a yes/no format, which can trigger social desirability bias. Instead, we offered a range of response options—for example, “I don't know, but I am worried” and “Yes, but I am not currently receiving treatment.” This approach allowed adolescents to express uncertainty or concern without requiring them to self‐identify as having a mental illness. Consequently, students might have felt comfortable disclosing emotional difficulties, which explains the relatively high prevalence of reported mental health concerns despite potential stigma.

Adolescents often turn to the internet as an easily accessible source for health‐related information [31]. Although internet sources are convenient, their quality and reliability can be inconsistent [32]. Therefore, encouraging adolescents to discuss health concerns with a trusted adult is crucial for ensuring that they receive accurate guidance and can effectively navigate the overwhelming amount of online information. Promoting this dialog is essential for improving health outcomes.

We found that adolescent boys were less likely to speak with a trusted adult when experiencing health concerns, which is consistent with previous study findings [33]. Boys are more likely than girls to face strong social pressure to suppress emotional expression and maintain independence, which reduces the likelihood of seeking help for physical or mental health concerns. Cultural expectations regarding masculinity may further discourage boys from acknowledging vulnerability, particularly in settings where open discussion on emotional or health‐related problems is stigmatized. Adolescents from urban areas were less likely to discuss health concerns with trusted adults than those from rural areas, possibly reflecting weaker interpersonal support, differences in family or community connectedness, or other urban–rural differences in help‐seeking patterns. Additionally, adolescents who consumed alcohol recreationally also tended to consult trusted adults more frequently. This could be specific to Mongolian culture, where drinking is a social tradition [34], promoting openness in discussing personal problems such as health concerns.

Our study has several limitations. First, participating schools were not randomly chosen, which may have introduced selection bias. However, the large sample size and diversity of school locations partially mitigate this problem. Second, online surveys may encounter reporting bias. Third, our measure of communication with a trusted adult referred to any of the listed health concerns rather than to a specific condition. Therefore, for adolescents reporting multiple concerns, we could not determine which particular concern they had discussed with an adult. Another limitation is that respondents who selected “Yes, but I am not currently receiving treatment” may not necessarily have required treatment for that condition. Therefore, this response should not be interpreted as definitive evidence of unmet clinical need.

## 5 Conclusion

Prompt management of physical health conditions is crucial for adolescents. The high prevalence of concerns regarding undiagnosed or untreated health conditions among adolescents can cause stress, highlighting the need for education that encourages them to share their concerns. Given the low likelihood of adolescents communicating their health concerns to trusted adults, school teachers and parents are encouraged to proactively initiate conversations and create supportive environments for open discussion.

## Author Contributions

**Ochirbat Batbold:** conceptualization, data curation, formal analysis, funding acquisition, project administration. **Baasanjav Nachin:** data curation, funding acquisition, resources. **Tserendolgor Lkhagvasuren:** data curation, funding acquisition. **Ser‐Od Erdenebileg:** data curation, funding acquisition. **Christy Pu:** conceptualization, formal analysis, writing – original draft, methodology, writing – review and editing.

## Ethics Statement

This study was approved by the Institutional Review Board of ACH Medical University (approval number: 20240722‐64). Informed consent has been appropriately obtained. Date of approval: 5/22/2024.

## Conflicts of Interest

The authors declare no conflicts of interest.

## Transparency Statement

Christy Pu affirms that this manuscript is an honest, accurate, and transparent account of the study being reported; that no important aspects of the study have been omitted; and that any discrepancies from the study as planned have been explained.

## Supporting information


Supporting File

## Data Availability

The data that support the findings of this study are available on request from the corresponding author. The data are not publicly available due to privacy or ethical restrictions.
