# Supplementary material for: Silent Worries: A Cross‐Sectional Study of School‐Going Adolescents' Unaddressed Health Concerns and Communication With Trusted Adults in Mongolia
Source: Health Sci Rep. 2026 Sep 13;9(9):e73025. doi: 10.1002/hsr2.73025 (PMC13572814; doi:10.1002/hsr2.73025)
Supplement: Supplementary file 1 [file HSR2-9-e73025-s001.docx]

| Supplementary Table S1. Combined prevalence of adolescents with perceived health concerns across the 12 health conditions | | | |
| --- | --- | --- | --- |
| Condition | Worried (%) | Untreated (%) | Combined (%) |
| Autism | 4.93 | 3.80 | 8.73 |
| Cancer | 5.32 | 3.00 | 8.32 |
| Musculoskeletal disorders | 5.60 | 4.55 | 10.15 |
| Asthma | 5.95 | 3.36 | 9.31 |
| Chronic respiratory tract disease | 6.20 | 4.72 | 10.92 |
| Hearing problem | 6.54 | 6.00 | 12.54 |
| Diabetes | 7.73 | 4.00 | 11.73 |
| Vision problem | 8.71 | 13.28 | 21.99 |
| Heart disease | 9.42 | 7.52 | 16.94 |
| Digestion problems | 11.23 | 11.19 | 22.42 |
| Depression | 12.09 | 15.49 | 27.58 |
| Anxiety | 12.11 | 15.49 | 27.60 |
